# Morganella morganii: A Rare Cause of Early-Onset Neonatal Sepsis

**DOI:** 10.7759/cureus.45600

**Published:** 2023-09-20

**Authors:** Inês Gameiro, Teresa Botelho, Ana Isabel Martins, Raquel Henriques, Patrícia Lapa

**Affiliations:** 1 Neonatology Department, Maternidade Daniel de Matos, Centro Hospitalar e Universitário de Coimbra, Coimbra, PRT

**Keywords:** bacteria, infectious diseases, newborn, neonatal sepsis, morganella morganii

## Abstract

*Morganella morganii* is an opportunistic Gram-negative bacillus commonly found in the human gastrointestinal tract and the environment. In adults, it is often associated with nosocomial infections, primarily surgical wound infections, urinary tract infections, and hepatobiliary infections. It is a rare cause of early-onset neonatal sepsis, with fewer than 15 reported cases in the literature. The authors aim to present a case of a low birth weight preterm born at 28 weeks' gestation, who developed early-onset neonatal sepsis due to *M. morganii*. We successfully treated the infection using a combination of third-generation cephalosporin and aminoglycoside, and in this report, we explain the rationale behind employing this antibiotic therapy.

## Introduction

Early-onset neonatal sepsis (EOS) presents as a multisystemic infection that occurs within the first three days of life and is associated with vertical transmission of microorganisms [1]. It can be caused by various agents [1], with *Morganella morganii *being a rare etiology of this infection [1-3].

*M. morganii* is a Gram-negative facultative anaerobic bacillus belonging to the Enterobacteriaceae family. It is an opportunistic microorganism found in the normal flora of the human gastrointestinal tract and the environment [1,3,4]. In adults, it is associated with nosocomial infections, mainly surgical wound infections, urinary tract infections (UTIs), and hepatobiliary infections; bacteremia is rare and often secondary [2,5,6].

*M. morganii* is an unusual infectious agent in the pediatric age group, especially in the neonatal period [2,7]. In addition to neonatal sepsis, it can rarely cause meningitis, pneumonia [5,6], necrotizing fasciitis, ventriculitis, cerebral abscesses, fetal death [6], and ocular infections [8].

The clinical manifestations of neonatal sepsis caused by *M. morganii* are nonspecific [1], and the most common antenatal risk factor is maternal chorioamnionitis. Other risk factors include premature rupture of membranes, maternal sepsis, and maternal UTIs caused by *M. morganii* during pregnancy [3,5]. This bacterium exhibits intrinsic resistance to most β-lactams, so unlike usual practice, third-generation cephalosporins are the first-line treatment for EOS caused by *M. morganii *[1,3]. The authors describe a case of EOS due to* M. morganii* and its progression, considering its peculiarities.

## Case presentation

A 36-year-old primigravida was admitted to the hospital at 25 weeks and six days gestation for preterm premature rupture of membranes. She was healthy, with an uneventful pregnancy until that date, with non-pathologic serologies (first trimester: HBs antigen, HIV-1/2 antibodies, VDRL, rubella IgG and IgM, toxoplasma IgG and IgM; second trimester: toxoplasma IgG and IgM) and normal ultrasounds. The mother received a complete antenatal steroid course with two doses of betamethasone given 24 hours apart. She started antibiotics according to local obstetrics protocol: intravenous ampicillin, gentamicin and azithromycin for three days and then five days of amoxicillin. During hospitalization, she presented mild leukocytosis with neutrophilia and negative C-reactive protein (CRP) level in repeated laboratory tests. Third-trimester serologies (HIV-1/2 antibodies, VDRL, toxoplasma IgG and IgM) were normal. She also had ultrasounds that showed a slightly decreased amniotic fluid index with a normal Doppler and 8/8 biophysical profile fetus. About 24 hours before delivery, she started clarithromycin due to positive vaginal exudate for *Ureaplasma urealyticum*.

A female neonate of 28 and 4/7 weeks gestational age was delivered by emergent cesarean section due to placental abruption with decelerations on cardiotocography. The birth weight was 1,200 g (very low birth weight, appropriate for gestational age). She was born hypotonic and bradycardic, requiring positive pressure ventilation and FiO_2_ of 0.3, and due to her low heart rate, she was intubated in the second minute of life (APGAR score 4, 7, and 8 at 1, 5, and 10 minutes, respectively). The neonate was admitted to the neonatal intensive care unit (NICU) and placed on synchronized intermittent positive pressure ventilation (SIPPV). Surfactant (200 mg/kg) was administered, an umbilical venous catheter was inserted, and septic screening was performed. Initial laboratory studies disclosed white blood cell count was 5.6 x 10^9^/L, with 0.6 x 10^9^/L (11.4%) neutrophils, 4.1 x 10^9^/L (73.6%) lymphocytes, and 165,000 /mL platelets. CRP levels were 4.5 mg/L. Hyperglycemia of 13.1 mmol/L was also identified, with an adjustment of glucose intake in the intravenous cocktail infusion. A combination of intravenous ampicillin (50 mg/kg/dose twice a day) and gentamicin (5 mg/kg/dose once daily) was started empirically after collecting a blood culture. On the second day of life, CRP levels peaked at 52.5 mg/L and the blood culture became positive. The newborn was extubated to continuous positive away pressure (CPAP) and presented hyperbilirubinemia of 130 µmol/L, starting phototherapy that was held for two days. On the third day of life, a lumbar puncture was performed, which was traumatic, with subsequent negative culture. She had digestive intolerance, characterized by bilious vomiting and abdominal distension, with improvement after a few hours of interruption of enteral nutrition. By the fourth day, the blood culture grew *M. morganii *susceptible in vitro to gentamicin and cephalosporins and resistant to amoxicillin and amoxicillin-clavulanate (Table 1). Antibiotic therapy was changed from ampicillin to ceftazidime (30 mg/kg/dose twice a day), while maintaining gentamicin, by guidance from the local infection committee. The next day, septic screening was repeated, with a resolution of neutropenia (white blood cells 9.9 x 10^9^/L, neutrophils 2.1 x 10^9^/L), and a decreasing trend in CRP levels (9.4 mg/L). *M. morganii* was also isolated in the culture from the tip of the endotracheal tube and the placenta. A total of 10 days of antibiotic therapy were completed.

**Table 1:** Antibiogram results

Antimicrobial agent	Result
Ampicillin	Resistant
Amoxicillin-clavulanate	Resistant
Ceftazidime	Susceptible
Meropenem	Susceptible
Gentamicin	Susceptible
Ciprofloxacin	Susceptible
Trimethoprim-sulfamethoxazole	Susceptible
Piperacillin-tazobactam	Susceptible

The patient remained stable throughout the hospitalization, breathing spontaneously since day 4, with the need for supplemental oxygen until day 33 (maximum FiO_2_ 0.3). Transfontanellar ultrasound initially showed bilateral parietal parenchymal hyperechogenicity, which later resolved. Stage 1 retinopathy of prematurity (ROP) was detected in the right eye on day 36. The patient was discharged on the 45th day of life (postmenstrual age 35 weeks) with a weight of 2,260 g (p72 on INTERGROWTH-21st charts). Follow-up appointments are being maintained. Cranial ultrasounds at four and six months were normal. Currently, at nine months of chronological age (corrected age six months), the patient is clinically well, without ROP, and with appropriate development.

## Discussion

Neonatal sepsis caused by *M. morganii *is rare, with only 15 cases described in the literature [9], most of which are early-onset [7]. Despite the limited literature due to the small number of cases, there was no sex predilection [1], with a median age of diagnosis at 1.3 days [4]. Almost all reported cases involve premature births [4,5], with approximately half being delivered by cesarean section [4], mirroring our own case.

It was possible to identify several risk factors, such as maternal chorioamnionitis (infection of the placenta) and premature rupture of membranes three weeks before. The presence of positive maternal cultures for *M. morganii *(urine, blood, placenta, vaginal discharge, and amniotic fluid) [1], as was the case with the placental culture in our situation, reinforces the notion of vertical transmission of this infection and vaginal colonization by this bacterium [9,10]. Similar to what has been described for other bacteria, the use of prophylactic antibiotics during pregnancy likely contributed to vaginal colonization by *M. morganii *and subsequent neonatal infection [11], as observed in this newborn.

The clinical manifestations of *M. morganii* sepsis, similar to neonatal sepsis from other etiologies, are nonspecific, including fever, tachycardia, hypotension, feeding difficulties, among others [1]. The most commonly reported findings were respiratory distress, tachypnea, and perinatal depression [9]. In the case mentioned, the presence of respiratory depression at birth, hyperglycemia and digestive intolerance were probable manifestations of sepsis. Although concomitant meningitis was not observed in our case, there have been four cases described in the literature [9], highlighting the importance of performing a lumbar puncture due to its implications in therapeutic management. Laboratory findings are also similar to other etiologies of neonatal sepsis, including leukocytosis, leukopenia, neutropenia, and increased levels of CRP in 50% of cases. Isolation of the microorganism in blood culture confirms the diagnosis [1].

As previously mentioned, *M. morganii* is resistant to ampicillin and amoxicillin, as demonstrated in this case, and many strains also exhibit reduced susceptibility to first and second-generation cephalosporins [3]. Therefore, the recommended treatment for uncomplicated bacteremia/sepsis involves the administration of third-generation cephalosporins ± an aminoglycoside for 10-14 days [1,4].

While it did not happen in this particular case and is relatively rare (described in less than 5% of *M. morganii* infections), it is important to remain vigilant about the possibility of resistance emerging during treatment with third-generation cephalosporins. *M. morganii *carries an inducible AmpC β-lactamase, which, when exposed to these antibiotics for an extended period, can result in its overproduction and subsequent development of resistance [12]. Therefore, it is recommended to repeat cultures early if there is no clinical or laboratory improvement, or if the condition worsens [10]. Despite the favorable clinical outcome in this neonate, EOS caused by *M. morganii* is associated with significant mortality (27%-40%) [3,4,10] and morbidity, especially in premature infants with low birth weight [1,4].

## Conclusions

This case highlights the importance of remaining aware of this rare pathogen, which can cause early-onset sepsis in neonates. *M. morganii* is generally resistant to commonly used beta-lactam antibiotics, although it usually demonstrates susceptibility to third-generation cephalosporins. Early recognition and appropriate antibiotic therapy guided by local epidemiology and confirmed sensitivity testing in neonates continue to be the optimal therapeutic approach.
